# A Rare Presentation of Becker’s Nevus on the Lower Extremity With Dermoscopic Features: A Case Report

**DOI:** 10.7759/cureus.67440

**Published:** 2024-08-21

**Authors:** Kerem Balan, Mehmet Alperen Lökoğlu, Başak Yalıcı Armağan

**Affiliations:** 1 Dermatology, Hacettepe University, Ankara, TUR

**Keywords:** puberty, lower extremity, hamartoma, dermoscopy, becker's nevus

## Abstract

Becker's nevus (BN) is a unilateral epidermal hamartoma that presents as a hyperpigmented and hypertrichotic lesion, typically appearing during adolescence. While BN frequently occurs on the upper trunk and proximal upper limbs, its manifestation on the lower limb is rare. Dermoscopy serves as a helpful diagnostic tool alongside clinical examination, revealing features such as pigment networks, hypertrophic follicles, and distinct skin furrows. We presented a case of a 17-year-old boy with typical dermoscopic features of BN located on the lower extremity. Although there is no definitive treatment for BN, most therapeutic interventions are primarily aimed at improving cosmetic appearance. In cases like ours, where the patient is not concerned about the cosmetic aspect, treatment may not be necessary. Laser therapies, in particular, have been shown to be effective in treating BN. There are no reported cases of malignant transformation in the literature. While associations with conditions such as malignant melanoma, vitiligo, and various skin appendages have been documented in case reports, BN is generally considered a benign clinical condition in most patients.

## Introduction

Becker's nevus (BN) is a hyperpigmented benign cutaneous hamartoma with both epidermal and dermal components [1-2]. The lesions are frequently accompanied by hypertrichosis, and the presence of satellite lesions around the main lesion is one of its characteristic features [3]. The incidence in males is three to five times higher than in females [4-5]. The presence of acne vulgaris around the lesion in some patients, along with the male predominance, suggests that androgenic stimulation may play a role in the development of BN [3]. Although the onset age of BN is around 6.6 (0-17) years, the average age at diagnosis is typically 19.4 (two to 55) years. In some cases, BN has been associated with developmental anomalies, as seen in BN syndrome [4]. While there have been reports of associations with dermatological conditions, such as vitiligo, lichen planus, basal cell carcinoma, and malignant melanoma, these occurrences are rare, and BN is generally regarded as a benign condition [6-8]. BN is most commonly found on the upper trunk, with reports of cases involving the lower extremities being notably rare. Some authors believe this rarity is due to underreporting [9]. Herein, we present a case of BN on the lower extremity in a 17-year-old patient who came to our clinic with a lesion that had been present for one year.

## Case presentation

**Figure 1:**
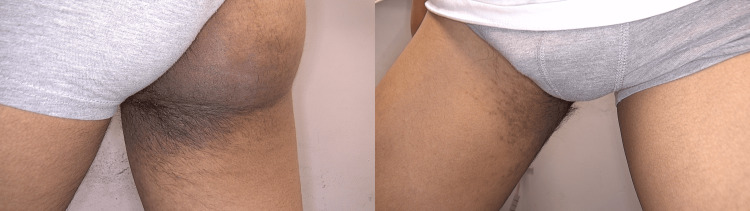
Hyperpigmented and hypertrichotic patch with satellite lesions on the buttock and anterior thigh

**Figure 2:**
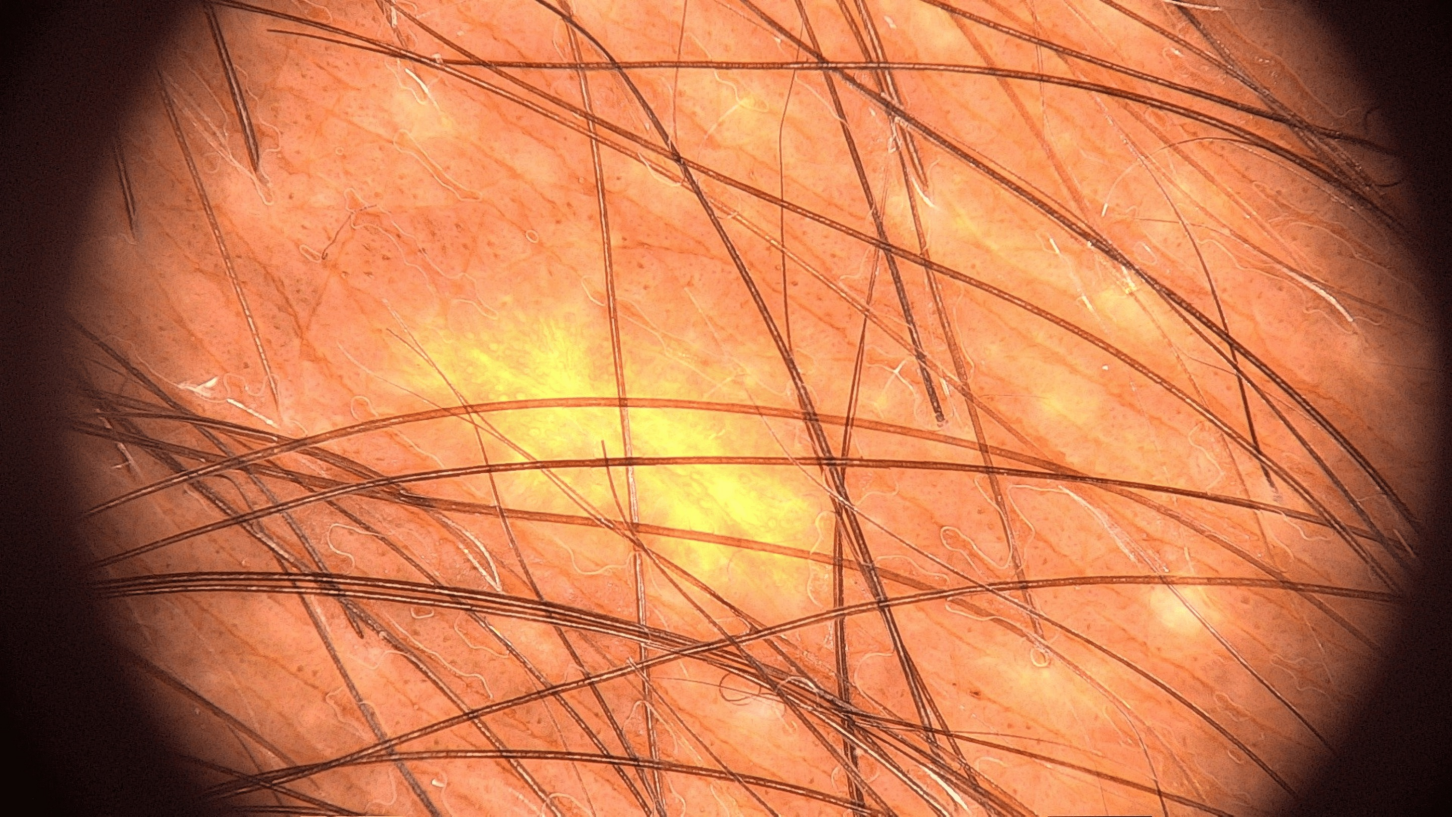
Thickening of the skin lines, perifollicular hypopigmentation, and the presence of brown dots

A 17-year-old healthy male patient presented to our clinic with concerns about a lesion located on the medial thigh and buttock (Figure 1). The patient reported that the lesion had been present for approximately one year. Importantly, there was no family history of skin cancer or other dermatological conditions, and no family members had a history of similar lesions. During the physical examination, a hyperpigmented patch was noted, characterized by terminal hairs and satellite lesions. This patch extended from the medial aspect of the right thigh to the gluteal region. The lesion was confined to this area, specifically extending from the buttock to the anterior thigh, with no involvement of other body parts. A thorough dermatological examination revealed no additional pathological findings elsewhere on the body. Further evaluation of the patient’s developmental milestones confirmed that they were normal, and no skeletal deformities or structural anomalies were detected through additional investigation. Dermoscopic analysis of the lesion revealed several distinct features: thickening of the skin lines, perifollicular hypopigmentation, and the presence of brown dots (Figure 2). Systemic examination did not reveal any other anomalies or health issues. Based on both the clinical examination and the dermoscopic findings, the lesion was diagnosed as BN. Although cosmetic laser treatment was proposed to potentially improve the appearance of the lesion, the patient declined this option. He expressed that he was not bothered by the cosmetic appearance of the lesion and thus chose not to pursue any treatment.

## Discussion

BN is typically a sporadic condition, with an overall prevalence estimated at around 1%. However, occasional familial cases have been reported. BN generally manifests around puberty, although both congenital and late-onset forms have been documented in the literature. The condition predominantly affects young males and is most commonly found on the upper trunk and proximal upper extremities. Involvement of the lower limbs is relatively rare, occurring in approximately 3% of cases. Most of these lesions are localized and typically do not extend beyond the knee [9]. The first case of BN on the lower extremity was reported in 1996 [10]. Notably, among the reported cases of BN on the lower extremities, three instances were associated with lipoatrophy [11-12]. The diagnosis of BN is primarily based on clinical examination; however, dermoscopy can provide valuable additional information. Key dermoscopic features of BN include a pigment network, perifollicular and focal hypopigmentation, hypertrophic hair follicles, thickened skin furrows, and the presence of vessels. These characteristics aid in differentiating BN from other similar dermatological conditions [13]. Treatment for BN is usually pursued for cosmetic reasons, as the condition itself is benign. The main therapeutic goals are to address hypertrichosis and hyperpigmentation associated with the lesion. Laser treatments have demonstrated considerable efficacy in managing these aspects of BN. Additionally, although the body of research is limited, some studies have indicated that topical treatments with antiandrogenic properties, such as flutamide and 70% glycolic acid, may offer partial benefits. Despite these findings, further research is needed to establish more comprehensive treatment protocols and evaluate the long-term outcomes of these interventions [3].

## Conclusions

BN is a benign cutaneous hamartoma typically found in young males. Although it is most commonly located on the upper parts of the body, such as the trunk and shoulders, its occurrence in the lower extremities should not be disregarded. While generally benign, it can be associated with developmental anomalies, and patients should be evaluated accordingly. Although treatment is usually pursued for cosmetic reasons, patients have been reported to benefit significantly from laser therapy. Despite its rarity in the lower extremities, this may be due to underreporting or patients not seeking treatment.
